# Pharmacy Technician-Administered Vaccines: On Perceptions and Practice Reality

**DOI:** 10.3390/pharmacy6040124

**Published:** 2018-11-29

**Authors:** Alex J. Adams, Shane P. Desselle, Kimberly C. McKeirnan

**Affiliations:** 1 Idaho State Board of Pharmacy, Boise, ID 83646, USA; 2 College of Pharmacy, Touro University California, 1310 Club Dr., Vallejo, CA 94592, USA; shane.desselle@tu.edu; 3 Center for Pharmacy Practice Research, College of Pharmacy and Pharmaceutical Sciences, Washington State University, Spokane, WA 99210, USA; Kimberly.mckeirnan@wsu.edu

**Keywords:** pharmacy technicians, immunizations, clinical pharmacy

## Abstract

Doucette and Schommer recently surveyed U.S. community pharmacy technicians on their willingness to perform tasks including the administration of vaccines. They found that 47.1% of technicians reported they were “unwilling” to administer a vaccine, although this finding must be placed into proper context. The first nationwide survey of U.S. pharmacist perceptions on immunizations in 1998 revealed only 2.2% of pharmacist respondents had administered adult vaccines and only 0.9% had administered childhood vaccines. They also found pharmacists to be “slightly negative on administering immunizations” with many perceived barriers. Nonetheless, pharmacist-provided immunizations have been an unqualified public health success. The theory of planned behavior (TPB) predicts intention from attitude and perceived behavioral control, among other factors. Given low involvement, exposure, and perceived behavioral control to administer vaccinations, technicians’ attitudes or willingness to participate from the Doucette and Shommer study can be regarded as quite positive. Given the results of a successful pilot project in Idaho and that subjective norms and perceived behavioral control will likely shift upward, one can only expect technicians’ willingness to participate in vaccinations to become more favorable and ultimately become a success.

The role of pharmacy technicians continues to advance globally. Pharmacy technicians are taking on broader roles with both medication dispensing support and clinical service support. The pharmacy profession in the United States (U.S.) faces the challenge of having national education standards and increasingly regional and national employers of technicians while each individual state determines what tasks pharmacy technicians can perform in their jurisdiction.

Recently, several U.S. states have considered public policy changes to allow pharmacy technicians to administer vaccines under the supervision of a pharmacist. While the administration of the vaccine does not have to be performed under the direct supervision of the pharmacist, generally the pharmacist must be physically present to assess the patient for the appropriateness of vaccine prescription and to assist with a response to a rare adverse event should it occur. Idaho became the first state to make this accommodation in 2017 [1,2]. Rhode Island followed in October 2018, and Utah is poised to become the third state to allow this activity by the year’s end. Several other states are in various stages of considering a law change for future years. As additional states consider this task, regulators will naturally look to the literature to determine the appropriateness of allowing their technicians to perform vaccine administration.

Doucette and Schommer recently surveyed U.S. community pharmacy technicians on their experience performing certain tasks, including vaccinations [3]. They found that while 18.9% of technicians reported they were “regularly involved” in preparing vaccinations for administration by a pharmacist, only 1.7% reported the same for administering a vaccination to a patient. While the literature has described successful technician involvement in various aspects of vaccine preparation (e.g., selecting the proper needle, loading the syringe, etc.), only one state allowed technicians to perform the technical task of vaccine administration at the time their survey was administered. As such, the results of the Doucette and Schommer study come as little surprise.

The authors also assessed technicians’ willingness to perform these same tasks [3]. They found that 47.1% of technicians reported they were “unwilling” to administer a vaccine and reported this to be “low” in willingness. However, that finding must be placed into the proper context. First and most obviously, the vast majority of respondents were not yet involved in this task, had not yet received any training to administer immunizations, and were practicing in states where doing so was not yet permissible by law. Indeed, survey respondents reported a higher uptake and higher willingness to perform tasks that have been allowed in a broader number of states, such as taking telephone prescription orders and performing final prescription verification during dispensing [4,5].

Another published study indicated low involvement by technicians in immunizations; however, that same study reported moderate attitudes and self-efficacies for taking part in immunizations [6]. The theory of planned behavior (TPB) predicts intention from attitude and perceived behavioral control, among other factors [7]. Given low involvement, exposure, and perceived behavioral control to administer vaccinations, technicians’ attitudes or willingness to participate from the Doucette and Shommer study can be regarded as positive.

As various U.S. regulators are actively considering allowing technicians to administer vaccines in their jurisdictions, some may question how these perceptions of willingness should influence policymakers, if at all, in deciding whether or not to allow technicians to perform this duty. To that end, it is useful to compare the results of this technician perception study to early studies on pharmacist perceptions of their own willingness to administer vaccines.

Madhavan and colleagues conducted the first nationwide survey of U.S. pharmacist perceptions on immunizations [8]. The survey was administered in 1998, four years after the first state allowed pharmacists to administer vaccines, and during a time when the number of states allowing pharmacists to perform this activity was increasing rapidly. The authors found only 2.2% of pharmacist respondents had administered adult vaccines and only 0.9% had administered childhood vaccines. It was more common for respondents to have worked in a pharmacy that brought in temporary nurses to provide vaccine clinics for adult patients (16.2%). They also found pharmacists to be “slightly negative on administering immunizations” with oft-cited barriers of (1) lack of time, (2) concern about legal liability, and (3) level of reimbursement.

If policymakers had used the early pharmacist survey results to determine whether pharmacists should administer vaccines, they would not have concluded in the affirmative. Foregone would be the significant public health outcomes that have since been achieved, including increased vaccination rates, high rates of patient satisfaction, and lower costs when compared to vaccines provided in other settings [9,10,11]. Pharmacist immunizations were highlighted as a major success story during the 2009 H1N1 influenza pandemic, and federal public health officials have now embedded pharmacists into future pandemic preparedness scenarios given the convenient infrastructure they offer [12]. Delayed policymaking based on initial pharmacist ambivalence would have come at a significant cost to patients and to the profession.

As Charrois recently noted, perception studies ultimately assess opinions that are “value-laden and biased, and do not necessarily relate to optimal patient care.” [13]. These issues are particularly acute when perceptions are assessed from other health professions that may be construed as market competitors. As it relates to immunizations, 46% of Canadian physicians and 32% of nurses “strongly disagreed” with allowing pharmacists to immunize [14]. As a result, studies of perceptions, particularly those undertaken prior to respondent exposure, must be taken in context and in light of considerable limitations, especially in reference to policy-making.

Such studies may, however, have greater utility from a practice perspective so as to guide the implementation of a new service [15]. For example, Madhaven found that pharmacists’ willingness to administer vaccines increased in the subgroup of pharmacists who had attended an immunization training program. This finding spoke to the benefit of having robust pharmacist training, and more than 300,000 pharmacists have since completed a skill-specific certificate training program [16]. The accreditation standards for entry-level Doctor of Pharmacy graduates now embeds this training into the curriculum of all U.S. schools and colleges of pharmacy. The finding and subsequent outcomes speak to the usefulness of the TPB. Outside the U.S., a recent study of pharmaconomists in Denmark also found a gap between attitude and self-efficacy; that is, they were more positive about the prospects than they were confident in the ability to administer immunization because they had not yet begun to do so. At the same time, however, their professional organizations continue to lobby for a greater scope of practice and demonstration projects to accommodate this possibility [17]. Similar results have been demonstrated for various technician activities in New Zealand and elsewhere [18].

A similar pathway can be envisioned for the inclusion of pharmacy technicians to administer vaccinations. A study on the first program to train pharmacy technicians on proper vaccine administration technique described how, following the completion of a home study and live training program, technicians self-reported an increased confidence with vaccine administration skills [19]. These technicians went on to successfully administer 953 immunizations without issue. The program has since expanded, along with at least two other training programs, and to date more than 300 Idaho technicians have completed vaccine administration training. An estimated 25,000 vaccines having been administered by Idaho technicians and no adverse events or errors reported to the state’s Board of Pharmacy.

In general, public policy should be established based on the public interest. The experience in Idaho lends credence to the strong safety profile that has accompanied pharmacy-technician-administered vaccines. This track record is of little surprise, as technicians have a similar educational background to other health professions (namely, medical assistants) that have administered vaccines for years under the supervision of physicians. Further, states continue to explore opportunities to transition to a more permissive “standard of care” approach to regulation that lends itself to all members of the pharmacy team practicing to the full extent of their clinical ability [20]. Thus, additional states should remove their regulatory restrictions and allow properly trained technicians to administer vaccines in the years ahead.
